# Factors predicting death and cancer in patients with giant cell arteritis in Western Norway 1972–2012: a retrospective observational cohort study

**DOI:** 10.3389/fmed.2023.1243791

**Published:** 2023-09-08

**Authors:** Lene Kristin Brekke, Jörg Assmus, Bjørg-Tilde Svanes Fevang

**Affiliations:** ^1^Department of Rheumatology, Haugesund Hospital for Rheumatic Diseases, Haugesund, Norway; ^2^Bergen Group of Epidemiology and Biomarkers in Rheumatic Disease (BEaBIRD), Department of Rheumatology, Haukeland University Hospital, Bergen, Norway; ^3^Centre for Clinical Research, Haukeland University Hospital, Bergen, Norway; ^4^Department of Clinical Science, University of Bergen, Bergen, Norway

**Keywords:** vasculitis, giant cell arteritis, temporal arteritis, epidemiology, death, cancer

## Abstract

**Objectives:**

Evidence as to whether or not giant cell arteritis (GCA) confers added risk of cancer or death is conflicting. Our aim was to identify factors predicting death or cancer in a large Norwegian GCA-cohort.

**Methods:**

This is a retrospective observational cohort study including patients diagnosed with GCA in Western Norway during 1972–2012. Patients were identified through computerized hospital records using the International Classification of Diseases coding. Medical records were reviewed and data about registered deaths and cancer occurrences were extracted from the Norwegian Cause of Death Registry and the Cancer Registry of Norway. We investigated predicting factors using Cox proportional hazards regression.

**Results:**

We identified 881 cases with a validated diagnosis of GCA (60% biopsy-verified). 490 patients (56%) died during the study period. Among 767 patients with no registered cancer prior to GCA diagnosis, 120 (16%) were diagnosed with cancer during the study period. Traditional risk factors were the main predictors of death; age at time of GCA-diagnosis [hazard ratio (HR) 2.81], smoking (HR 1.61), hypertension (HR 1.48) and previous cardiovascular disease (HR 1.26). Hemoglobin (Hb) level was also associated with risk of death with increasing Hb-levels at time of GCA-diagnosis indicating decreased risk of death (HR 0.91). Other GCA-related factors were not predictive of death. We did not identify any predictors of cancer risk.

**Conclusion:**

In our cohort of GCA-patients, the risk of death was predominantly predicted by age and traditional risk factors. We found no significant associations with regards to the risk of incident cancer.

## Introduction

Giant cell arteritis (GCA) is the most common systemic vasculitis in adults. It is almost exclusively a disease of persons older than 50 years, with peak onset between 70 and 80 years. The population aging trend is most advanced in Europe and Northern America, where also the highest incidence of GCA has been reported. Further increase in life expectancy will affect both cancer-and GCA-epidemiology. The pathogenesis of GCA is recognized as a widespread inflammatory state affecting large and medium-sized arteries, and irreversible ischemic complications may follow. Furthermore, chronic inflammation is a well-documented risk factor for cancer (1). Nevertheless, evidence as to whether or not GCA confers added risk of cancer or death is conflicting and the understanding of contributing risks is incomplete (2–7). A major challenge for investigators has been lack of or limited ability to adjust for confounders, and limitations due to small sample sizes and/or short periods of follow-up. We report a 41-year follow-up study of 881 patients with validated GCA-diagnoses for whom multiple candidate risk factors were assessable. This study aims to investigate possible individual risk factors for cancer and death within the GCA-population.

## Methods

This is a retrospective cohort study including patients diagnosed with GCA in Bergen Health Area (Norway) during 1972–2012. Patients were recruited from three somatic hospitals: Haukeland University Hospital, Haraldsplass Deaconess Hospital and Voss Hospital. The population size in the uptake area of these hospitals is approximately 440,000 inhabitants. Patients were identified through computerized hospital records using the International Classification of Diseases coding system. We collected data by reviewing medical records of all patients registered with a GCA-diagnosis following an outpatient visit or admission to any ward in the study hospitals from January 1st in 1972 until the end of study December 31st in 2012. We use the term “validated diagnosis” to indicate that an expert (rheumatologist), following chart review, agreed that clinical information was consistent with the diagnosis of GCA and not more likely to represent another disease. Clinical features including laboratory findings and histology were registered at the time of diagnosis. The status for traditional risk factors were also, and only, registered at time of diagnosis, i.e., upon entry into the cohort (baseline). Pharmacologic treatment (dose and type) was registered based on hospital documentation with extracted variables limited to the starting-dose and maximal dose of prednisolone prior to first registered remission or end of study. The observation period ended when the patient died or when the study ended. Further details about the inclusion process and characteristics of the GCA-population have been published previously (8). Extensive demographic and clinical data were collected. If vasculitis-related symptoms or clinical findings we sought were not mentioned in the medical records, they were considered absent. If laboratory or imaging parameters were missing they were registered as missing in the data set. To ensure accuracy and completeness of demographic and clinical data not related to the vasculitis (i.e., date/type of cancer and date/cause of death), information on these variables was obtained from national registries. We thus had available data allowing risk estimates for the following variables: age at GCA-onset (years, continuous variable), sex (male/female), centrality (urban/rural), year of diagnosis (categorized into 1972–1982, 1983–1992, 1993–2002, or 2003–2012), smoking status (yes/no/unknown), pre-existing diabetes (yes/no), pre-existing hypertension (yes/no), previous cardiovascular disease (yes/no), temporal artery biopsy (positive/negative), giant cells in biopsy (yes/no), polymyalgia rheumatica (PMR; present/absent), jaw claudication (present/absent), visual disturbance (any type assumed related to arteritis – yes/no), blindness (assumed related to GCA – yes/no), temporal artery tenderness or reduced pulsation (yes/no), C-reactive protein (CRP, mg/L), erythrocyte sedimentation rate (ESR, mm/h), hemoglobin (Hb, g/dL), white cell count (x10^9^/L), platelets (x10^9^/L), initial prednisolone-dose (i.e., first dose following GCA-diagnosis, mg/day), maximum prednisolone-dose (i.e., prednisolone-dose before first attempt at tapering, mg/day). Unfortunately, large vessel (LV) involvement was not systematically documented in the time period of our study. Data, based on medical imaging technologies, on LV involvement was available for <1% of the cohort. Hence, this variable could not be included, nor could we attain data on complete duration of steroid treatment or cumulative steroid dose as late follow-up was partly done in general practice and data thus unavailable. Date of deaths were obtained from the Norwegian Cause of Death Registry to which the death of every Norwegian is mandatorily reported. Date and type of incident cancers were obtained from the Cancer Registry of Norway, in which all new cases of cancer in Norway (except basal cell carcinomas) have been registered since 1952. For cancer-analyses, we excluded patients with registered cancer diagnosis prior to GCA-diagnosis. This study was approved by REK sør-øst B regional ethics committee (study reference number 2012/643/REK sør-øst B).

### Statistical analysis

We investigated predicting factors for hazard of death or cancer within the GCA-cohort using Cox proportional hazards regression. Selected variables were first analyzed in univariate and block regression models (block 1: clinical features including histology, block 2: laboratory and treatment factors, block 3: demographic and traditional risk factors). Variables included in the final multivariable model were selected on the following basis: value of *p* < 0.1 in univariate or block regression or otherwise deemed clinically relevant. For the analysis concerning hazard of death all parameters included in the final multivariable model were selected based on results (value of *p* < 0.1) in uni- or block analysis. For the analysis concerning hazard of cancer the parameters “age at GCA diagnosis” and “year of diagnosis” were included in the multivariable regression due to presumed clinical relevance despite value of *p* > 0.1 in uni- and block analyses. To minimize the risk of variable structure impacting the findings we kept “unknown,” in addition to “yes” and “no,” as possible values for the predictor “smoking status.” Though presumed to have high clinical relevance, the variable “LV involvement” was omitted from the analyses due to a high degree of missing data. Significance level in the final model was set to 0.05. Computing was done using SPSS version 26 (IBM Corp, Armonk) and R 4.0 (9). Graphics were created using Matlab 9.0 (Mathworks Inc., Natick).

## Results

**Figure 1:**
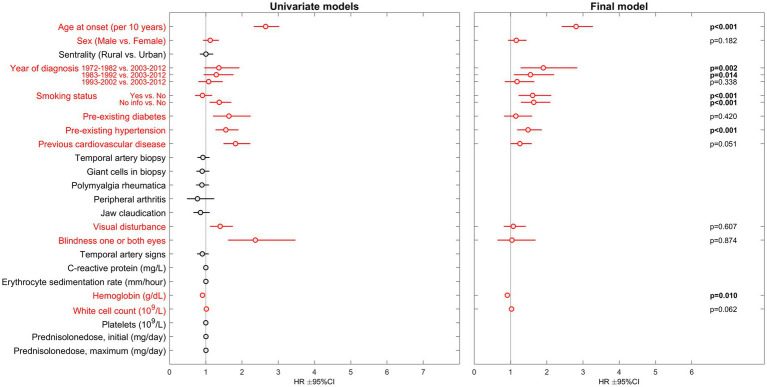
Results of Cox proportional hazards regression regarding risk of death in GCA-patients. HR, hazard ratio; CI, confidence interval. Parameters included in the multivariable analysis are presented in red.

A total of 881 patients (71% female) were included following validation of the GCA-diagnosis. Mean age was 73 years (SD 9). 490 patients (56%) died during the study period. In final multivariable analysis, the traditional risk factors were most strongly associated with hazard of death: age at onset of GCA (HR 2.81, 95% CI 2.41–3.27, *p* < 0.01), smoking (HR 1.61, 95% CI 1.22–2.12, *p* < 0.01), hypertension (HR 1.48, 95% CI 1.18–1.86, *p* < 0.01) and previous cardiovascular disease (HR 1.26, 95% CI 1.00–1.58, *p* = 0.05). Among laboratory parameters only Hemoglobin (Hb) levels were significantly associated with hazard of death with increasing Hb-levels indicating decreased risk (HR 0.91, 95% CI 0.85–0.98, *p* = 0.01). Presenting with visual disturbance (any) was associated with increased hazard of death in univariate analysis (HR 1.40, 95% CI 1.12–1.75, *p* < 0.01). However, this association was not observed in the multivariable analyses. Complete results from univariate and the final multivariable model are presented in Figure 1.

**Figure 2:**
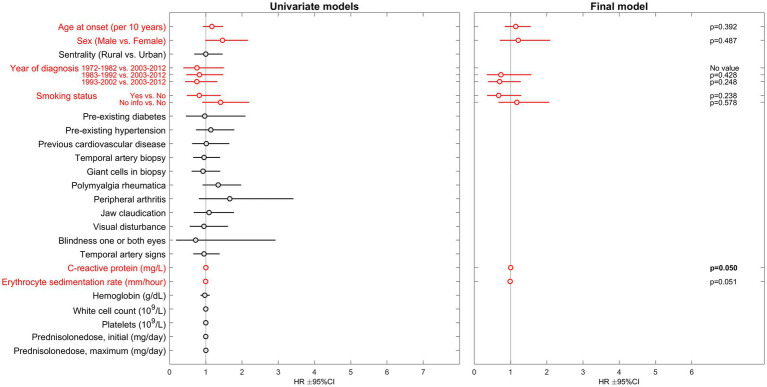
Results of Cox proportional hazards regression regarding risk of cancer in GCA-patients. HR, hazard ratio; CI, confidence interval. Parameters included in the multivariable analysis are presented in red.

Among the 767 patients (72% female) with no registered cancer prior to GCA diagnosis, 120 (16%) were diagnosed with a first cancer during the observation period. No investigated variable was significantly associated with hazard of cancer in the final multivariable model (Figure 2). The complete uni-, block- and multivariable regression results are attached as supplementary material; Supplementary Table 1 for death analyses and Supplementary Table 2 for cancer analyses.

## Discussion

In our large cohort of GCA-patients, the risk of death was predominantly predicted by age at onset of GCA and traditional risk factors (smoking, hypertension and previous cardiovascular disease). These are common risk factors for cardiovascular events, which were the most frequent causes of death in this cohort (3). Cardiovascular disease has also been identified as the most frequent causes of death in other GCA-cohorts (2, 10–13). However, recent publications indicate that GCA-patients may have beneficial risk profiles with regards to some cardiovascular risk factors. A significant inverse relationship between body mass index and GCA was demonstrated in a meta-analysis published in 2015 (14). More recently, Wadström et al. documented that GCA-patients had significantly lower fasting blood glucose, cholesterol and triglycerides compared to controls (15). These apparent contradictions challenge our understanding of what actually causes cardiovascular deaths in GCA-patients. Cardiovascular disease risk is multifactorial and contribution of one risk factor may be difficult to quantify independently of other factors. Furthermore, to our knowledge, robust data on the risk attributable to factors with great complexity, such as diet, physical activity patterns and socio-economic status are hitherto non-existent in the context of GCA. Furthermore, there is an inevitable connection for both carcinogenesis and mortality with increasing age. The exponential increase in death risk with chronological age is often referred to as the rate-of-aging. A recent study exploring whether the onset of severe chronic disease alters the rate-of-aging concluded that the rate-of-aging process in itself is not affected by disease history, but rather an underlying process of aging that causes mortality to increase at a set pace (16). Our results may be viewed as support to this perception.

The widening clinical spectrum of GCA has been well described in recent years, but there are still no definitions for disease subsets other than rough categorization into pure cranial GCA, mixed cranial and LV GCA and purely non-cranial GCA. Data on the presence or absence of LV involvement was available for <1% of our sample. Hence, this variable could not be included in our analyses. However, more recent studies with improved stratification of disease subsets are limited by shorter follow-up and the possibility of incomplete capture of deaths due to late vascular complications.

Macchioni et al. reported that presenting with PMR was associated with reduced mortality risk (17). We were not able to confirm this finding. In fact, no GCA-specific clinical feature was significantly associated with death in our multivariable model. Visual disturbance (HR 1.40), and visual loss (HR 2.37), were associated with risk of death in univariate analyses. Few events may explain the lack of association in our multivariable analysis. No specific GCA-symptom, finding, laboratory-parameter or treatment factor were found to be predictive of cancer risk in our GCA-cohort. This is in line with our previous finding of similar cancer risk in GCA-patients compared to population controls (7). However, our analyses could not confirm the known association of cancer with advancing age and smoking. This may be due to small numbers of events (120 incident cancers in 41 years).

### Limitations

Our data are limited by the retrospective design and lack of information about LV involvement and cumulative steroid burden. A power analysis was not performed and weaker associations, or associations pertaining only to subgroups, may be undetected. Competing risk analysis was not performed in this work despite death being a competing risk when performing Cox analysis with cancer as the outcome of interest. Furthermore, some potential risk factors were not accounted for (e.g., diet, body mass, activity patterns, and sun exposure). However, a major strength of our study is the large cohort of patients with validated GCA-diagnoses resulting from thorough review of clinical data and exclusion of misclassified cases. Furthermore, the long follow-up period is vital when studying potential late outcomes such as death and cancer. Access to national registries with mandatory reporting provided excellent completeness of data on cancer and death.

## Conclusion

Deaths in our cohort were predominantly predicted by modifiable cardiovascular risk factors in addition to age and existing co-morbidity. However, contributing risk factors for circulatory death needs to be further explored as this patient group is expected to expand along with the phenomenon of global aging.

## Data availability statement

The data analyzed in this study is subject to the following licenses/restrictions: data is not available for privacy reasons. Requests to access these datasets should be directed to lene.kristin.brekke@hsr.as.

## Ethics statement

The studies involving humans were approved by REK sør-øst B Regional Ethics Committee. The studies were conducted in accordance with the local legislation and institutional requirements. The ethics committee/institutional review board waived the requirement of written informed consent for participation from the participants or the participants’ legal guardians/next of kin because of the long duration of the study and the late onset of the disease.

## Author contributions

All authors were involved in drafting the article or revising it critically for intellectual content and approved the final version submitted for publication.

## Funding

This study was supported by unrestricted grants from the Norwegian Association of Heart and Lung Patients, the Norwegian Rheumatism Association, Marit Hansens Memorial Fund, Merck Sharp & Dohme, Odd Fellow Medical Research Fund, the Raagholt Foundation, and the Norwegian Women’s Public Health Association.

## Conflict of interest

The authors declare that the research was conducted in the absence of any commercial or financial relationships that could be construed as a potential conflict of interest.

## Publisher’s note

All claims expressed in this article are solely those of the authors and do not necessarily represent those of their affiliated organizations, or those of the publisher, the editors and the reviewers. Any product that may be evaluated in this article, or claim that may be made by its manufacturer, is not guaranteed or endorsed by the publisher.

## Supplementary material

SUPPLEMENTARY TABLE 1 Complete uni-, block and final multivariable regression results for mortality analysis. N, number of observations; HR, hazard ratio; CI, confidence interval.

SUPPLEMENTARY TABLE 2 Complete uni-, block and final multivariable regression results for cancer analysis. N, number of observations; HR, hazard ratio; CI, confidence interval.

## Abbreviations

CI: Confidence Interval
CRP: C-reactive Protein
ESR: Erythrocyte Sedimentation Rate
GCA: Giant Cell Arteritis
Hb: Hemoglobin
HR: Hazard Ratio
LV: Large Vessel
PMR: Polymyalgia Rheumatica
SD: Standard Deviation
SPSS: Statistical Package for the Social Sciences
